# Mapping the biomedical sciences using Medical Subject Headings: a comparison between MeSH co-assignments and MeSH citation pairs

**DOI:** 10.5195/jmla.2021.1173

**Published:** 2021-07-01

**Authors:** Fei Shu, Junping Qiu, Vincent Larivière

**Affiliations:** 1 fei.shu@mail.mcgill.ca, Professor, Chinese Academy of Science and Education Evaluation, Hangzhou Dianzi University, China; 2 casee.hdu@outlook.com, Professor, Chinese Academy of Science and Education Evaluation, Hangzhou Dianzi University, China; 3 vincent.lariviere@umontreal.ca, Professor, École de bibliothéconomie et des sciences de l'information, Université de Montréal, Canada

**Keywords:** MeSH, citation, co-assignment, map of life sciences, Gephi

## Abstract

**Objective:**

This study compares two maps of biomedical sciences using Medical Subject Headings (MeSH) term co-assignments versus MeSH terms of citing/cited articles and reveals similarities and differences between the two approaches.

**Methods:**

MeSH terms assigned to 397,475 journal articles published in 2015, as well as their 4,632,992 cited references, were retrieved from Web of Science and MEDLINE databases, respectively, which formed over 7 million MeSH co-assignments and nearly 18 million direct citation pairs. We generated six network visualizations of biomedical science at three levels using Gephi software based on these MeSH co-assignments and citation pairs.

**Results:**

The MeSH co-assignment map contained more nodes and edges, as MeSH co-assignments cover all medical topics discussed in articles. By contrast, the MeSH citation map contained fewer but larger nodes and wider edges, as citation links indicate connections to two similar medical topics.

**Conclusion:**

These two types of maps emphasize different aspects of biomedical sciences, with MeSH co-assignment maps focusing on the relationship between topics in different categories and MeSH direct citation maps providing insights into relationships between topics in the same or similar category.

## INTRODUCTION

The purpose of science mapping is to visualize the structure of scientific inquiry [1, 2], which helps us understand the evolution of science [3, 4]. Such mapping is generally derived from the metadata of scholarly articles such as author names, journal titles, disciplines, and cited references [5]; these elements and their connections form the nodes and edges of networks that can be visualized as maps. Although citation analysis is the dominant method for generating maps of science, other types of information could also be used, such as subject categories, research topics, course descriptions, or subject headings. For instance, expert judgment was first used for mapping science when Bernal drew, by hand, a map of science representing the hierarchical structure of scientific topics [6]. Small and Griffith then created the first citation-based map of science using co-citation analysis [7]. Since then, citation analyses—including direct citation, bibliography coupling, and co-citation—have been widely used for mapping science.

More recently, other methods have also been used for mapping science. For example, maps of science have been generated based on the co-occurrence of words in titles, abstracts, or keywords using coword analysis [8–12]. Balaban and Klein mapped science using undergraduate course prerequisites at Texas A&M University [13]. Suominen and Toivanen generated a map of science using topic modeling based on latent patterns in texts retrieved from Web of Science (WoS) [14]. Also, Shu et al. produced a map of science based on nonfiction books and their Library of Congress Subject Headings (LCSH) co-assignments [15].

The Medical Subject Headings (MeSH) thesaurus, created and maintained by the National Library of Medicine, is used in MEDLINE/PubMed and other biomedical databases and archives. Around 61,000 MeSH terms representing medical topics—from broad to specific—are organized in a hierarchical tree covering sixteen branches that can reach up to fourteen levels of depth. For example, *Organisms* is classified as a level 1 MeSH term (category B), *Aedes* is classified as a level 14 MeSH term, and the hierarchical structure *Organisms/Eukaryota/Animals/Invertebrates/Arthropods/Insecta/Pterygota/Neoptera/Holometabola/Diptera/Nematocera/Culicomorpha/Culicidae/Aedes* represents a branch from the broadest term *Organisms* to the narrowest term *Aedes.*

As biomedical science is the largest portion of the sciences, most current mapping approaches have been used to map biomedical science as a subset of the map of science. However, few studies apply PubMed's MeSH to the map of biomedical sciences. Leydesdorff et al. produced a base map using the MeSH categories C (Diseases), D (Chemicals and Drugs), and E (Analytical, Diagnostic and Therapeutic Techniques and Equipment) [16]. Leydesdorff et al. compared MeSH terms with cited sources among the literature related to Alzheimer disease and found that citations indicate the core structure of research, whereas MeSH terms represent relevance to current research options [17].

This study aims to contribute to the literature on science mapping by using MeSH [18] to present the structure and evolution of biomedical sciences. To compare this new mapping approach with the traditional citation-based mapping approach, we produced two maps using MeSH term co-assignments and MeSH terms of citing and cited papers, respectively. As scholarly documents in MEDLINE/PubMed can be assigned multiple MeSH terms, MeSH co-assignments express the likelihood that two medical topics are covered in the same, which allows mapping of the structure and evolution of biomedical sciences. Although this is a promising approach [5], no studies have yet generated a MeSH co-assignment–based map of biomedical sciences. Thus this study seeks to answer the following research question: How does a MeSH co-assignment map of biomedical sciences differ from a direct citation-based map using MeSH terms as controlled topics?

## METHODS

In MEDLINE/PubMed, each medical journal article is indexed with around ten to fifteen MeSH terms representing all topics related to or discussed substantially in the article [19]. Some of these assigned MeSH terms are designated as major, indicating an article's primary topics, whereas the others represent topics only discussed in the article. MeSH co-assignments can be used as a measure of the relative strength of the relationship between two MeSH terms, as these co-assignments express the likelihood that existing knowledge about two medical topics will be read together in the same article [15]. Thus a map of biomedical sciences can be generated on the basis of MeSH co-assignments.

A relationship between a citing article and its cited reference can also be established by a citation link. Thus a relationship between MeSH terms (representing medical topics) assigned to a citing article and its cited reference can be established by a citation, generating a traditional direct citation-based map using MeSH terms as controlled topics assigned to individual articles. This means that a MeSH-based map of biomedical sciences can be generated by two approaches: (1) using MeSH co-assignments within the same article, and (2) using MeSH terms assigned to a citing article and its cited references as controlled topics.

MeSH major topics are the major topics of the article, whereas non-major MeSH terms are usually related topics substantively discussed within the article [19]. The cooccurrence of MeSH major topics and their related non-major MeSH terms represents the relationship between two corresponding medical topics. Thus we used each co-assignment of two MeSH major topics or one MeSH major topic and one non-major MeSH term to generate the MeSH co-assignment map. In addition, considering that a citation represents the relationship between a citing article and its cited reference on the basis of relevant themes [20], we used the MeSH major topics of citing articles and their cited references to produce the MeSH direct citation map, representing their shared medical topics.

We retrieved 397,475 research articles published in 2015, as well as their 4,632,992 cited references from WoS. Only those cited references indexed by WoS were included in the dataset. All articles were classified as the discipline Clinical Medicine in the National Science Foundation (NSF) classification system, which is a two-level journal classification system consisting of fourteen broad fields and 144 subfields integrated into the WoS database. The NSF classification system exclusively assigns each individual journal into only one single field, as opposed to WoS categories, which assign journals to multiple categories.

Next, a version of MEDLINE/PubMed integrated into WoS was used as the linkage between WoS and PubMed, in which a PubMed unique article reference number (PMID) and MeSH terms were assigned to each journal article. As not all articles were covered by both WoS and PubMed, only citing articles and cited references with a PMID were included in this study. In total, 349,135 citing articles and their 1,899,457 cited references were included; 4,774,345 MeSH terms, including 276,677 major MeSH topics, were assigned to the citing articles, and 9,111,007 MeSH major topics were assigned to the cited references.

The maps generated in this study were based on the sixteen level 1 MeSH terms and 118 level 2 MeSH terms. Assigned MeSH terms at level 3 or lower were reassigned to their parent level 2 or grandparent level 1 MeSH terms. For example, for the hierarchical structure of *Organisms/Eukaryota/Animals/Invertebrates/Arthropods/Insecta/Pterygota/Neoptera/Holometabola/Diptera/Nematocera/Culicomorpha/Culicidae/Aedes,* the MeSH terms *Animals, Invertebrates, Arthropods, Insecta, Pterygota, Neoptera, Holometabola, Diptera, Nematocera, Culicomorpha, Culicidae,* and *Aedes* were reassigned to *Organisms* (level 1) or *Eukaryota* (level 2) when producing the MeSH co-assignment map at level 1 or 2. This method of reassignment to broader or more general abstraction levels has been used in previous studies of library classification mapping, which have confirmed its robustness [15].

Four datasets (4,325,056 MeSH co-assignments at level 1, 7,492,116 MeSH co-assignments at level 2, 10,071,906 citation pairs at level 1, and 17,921,730 citation pairs at level 2) were finalized to produce four maps of biomedical sciences: MeSH co-assignment maps at levels 1 and 2 and MeSH direct citation maps at levels 1 and 2. For each dataset, MeSH terms, as well as their co-assignments or MeSH citation pairs (i.e., MeSH major topics between citing articles and cited references), were imported into free graph-drawing software Gephi [21] to generate a visual map of biomedical sciences. Each MeSH term was a node (i.e., circle), whereas each MeSH co-assignment or MeSH citation pair was an edge (i.e., connecting line). The number of assignments of each MeSH term determined the size of a node, whereas the number of MeSH co-assignments or MeSH citation pairs determined the weight of an edge. Although Gephi does not support TXT or RIS files exported from WoS as do other visualization software (e.g., VOSviewer, Citespace, Bibexcel), it is the only software that has been used for mapping subject headings (i.e., LCSH) [15]. Therefore, we selected Gephi to visualize MeSH, which is similar to LCSH in terms of format and indexing. In addition to these four maps, we produced a co-assignment map and a direct citation map based on level 2 MeSH terms under category C (*Diseases*), as these terms represent the basic structure of biomedical science and were used for mapping in a previous study [16].

There is no strict rule regarding the selection of representative data for visualization [22], but thresholds have been frequently used in science mapping [23–25]. Based on the MeSH co-assignment and MeSH citation pair data, two threshold filters (number of MeSH co-assignments > 1,699, number of MeSH citation pairs > 699) were devised and respectively applied to two maps at level 2 to reduce them to a manageable number of visual elements. These thresholds produced two smallest subsets that account for at least 95% of MeSH co-assignments and MeSH citation pairs, respectively.

## RESULTS

### Level 1 map

**Figure 1:**
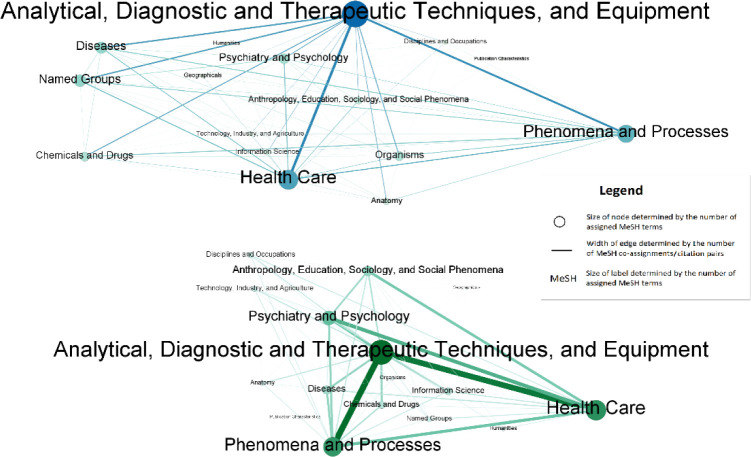
MeSH co-assignment map (upper) and MeSH direct citation map (lower) at level 1

Figure 1 shows two maps of biomedical sciences at the MeSH term level 1 containing sixteen blue nodes/110 edges (upper, co-assignment map) and sixteen green nodes/116 edges (lower, direct citation map). Nodes are level 1 MeSH terms, while edges represent their relationship (i.e., MeSH co-assignments and MeSH citation pairs, respectively). Edge width is proportional to the number of co-assignments or citation pairs between the two MeSH terms, and the node and label sizes are proportional to the number of assignments or citations. The shade of nodes/edges is also based on their size or width, as the color of large nodes or wide edges are darker.

For both the co-assignment map and the direct citation map, a strong triangular relationship among *Analytical, Diagnostic and Therapeutic Techniques and Equipment, Health Care,* and *Phenomena and Processes* was found. Indeed, as indicated in Table 1, seven out of the top ten MeSH terms (bold) in both maps were the same, with similar proportions of the total assigned MeSH terms. Some differences were observed; however, the nodes *Name Groups, Diseases,* and *Organisms* were relatively large in the co-assignment map but small in the direct citation map, and the node *Psychiatry and Psychology*, as well as its links, were stronger in the direct citation map than in the co-assignment map.

**Table 1:** Distribution of MeSH terms (level 1) in the co-assignment map and direct citation map

MeSH	% of assigned MeSH terms in the co-assignment map	% of assigned MeSH terms in the citation map
**Analytical, Diagnostic and Therapeutic Techniques, and Equipment**	21.05%	20.97%
**Health Care**	14.95%	16.73%
**Phenomena and Processes**	13.08%	15.75%
Named Groups	8.28%	2.63%
**Diseases**	8.19%	5.90%
**Psychiatry and Psychology**	6.75%	10.97%
**Chemicals and Drugs**	5.84%	4.32%
Organisms	5.83%	1.10%
**Anthropology, Education, Sociology, and Social Phenomena**	4.09%	7.17%
Anatomy	3.53%	2.21%
Information Science	2.71%	4.56%
Disciplines and Occupations	1.84%	3.14%
Technology, Industry, and Agriculture	1.81%	2.92%
Geographicals	1.27%	0.01%
Humanities	0.79%	1.62%
Publication Characteristics	<0.01%	<0.01%

### Level 2 map

**Figure 2:**
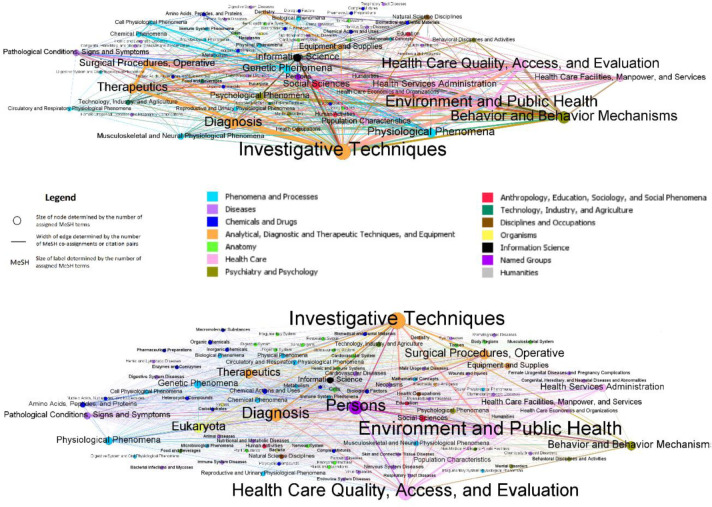
MeSH co-assignment map (upper) and MeSH direct citation map (lower) at level 2

Figure 2 shows two maps of biomedical sciences at the MeSH term level 2. In these maps, nodes are level 2 MeSH terms whose colors represent their parent MeSH terms at level 1, and the edge colors are mixed on the basis of the colors of their source nodes. Although the figure is visually complex due to high connectivity between nodes and overlapping edges, some differences were found when comparing the co-assignment map (left) and direct citation map (right). Whereas the distribution of MeSH citation pairs in the direct citation map was skewed, with some large nodes and wide edges, the distribution of MeSH co-assignments was more balanced. Indeed, the average degree (i.e., mean number of edges per node) and graph density (i.e., number of edges between nodes relative to the total possible number of edges between nodes) of the MeSH direct citation map (17.108 and 0.234, respectively) were lower than those of the MeSH co-assignment map (38.922 and 0.385, respectively).

When comparing the top 10 MeSH terms appearing in both maps, we found no major differences between maps (Table 2). Eight of the top ten MeSH terms were the same between maps; however, *Persons* and *Eukaryota* appeared more often in the MeSH co-assignment map, whereas *Genetic Phenomena* and *Information Science* appeared more often in the MeSH direct citation map.

**Table 2:** Top 10 MeSH terms in the level 2

MeSH	Parent MeSH (level 1)	% of assignments
Co-assignment map		
Investigative Techniques	Analytical, Diagnostic and Therapeutic Techniques and Equipment	6.65%
Environment and Public Health	Health Care	6.39%
Health Care Quality, Access, and Evaluation	Health Care	5.75%
Persons	Named Groups	5.29%
Diagnosis	Analytical, Diagnostic and Therapeutic Techniques and Equipment	4.90%
Eukaryota	Organisms	3.48%
Surgical Procedures, Operative	Analytical, Diagnostic and Therapeutic Techniques and Equipment	3.21%
Therapeutics	Analytical, Diagnostic and Therapeutic Techniques and Equipment	3.02%
Behavior and Behavior Mechanisms	Psychiatry and Psychology	2.83%
Physiological Phenomena	Phenomena and Processes	2.19%
Direct citation map		
Investigative Techniques	Analytical, Diagnostic and Therapeutic Techniques and Equipment	7.63%
Environment and Public Health	Health Care	5.94%
Behavior and Behavior Mechanisms	Psychiatry and Psychology	5.27%
Health Care Quality, Access, and Evaluation	Health Care	5.20%
Diagnosis	Analytical, Diagnostic and Therapeutic Techniques and Equipment	4.75%
Therapeutics	Analytical, Diagnostic and Therapeutic Techniques and Equipment	4.38%
Physiological Phenomena	Phenomena and Processes	3.61%
Surgical Procedures, Operative	Analytical, Diagnostic and Therapeutic Techniques and Equipment	3.08%
Genetic Phenomena	Phenomena and Processes	3.03%
Information Science	Information Science	2.82%

*Investigative Techniques* and *Environment and Public Health* were co-assigned or paired most often in both maps, and the top four MeSH co-assignments or citation pairs were the same in both maps (Table 3). However, *Persons* appeared in four out of the top 10 MeSH co-assignments in the co-assignment map, whereas *Behavior and Behavior Mechanisms* and *Therapeutics* and *Surgical Procedures, Operative* were only found in top 10 MeSH citation pairs in the direct citation map.

**Table 3:** Top 10 MeSH co-assignments and top 10 MeSH pairs in the level 2

MeSH 1	MeSH 2	% of co-assignment
Co-assignment map		
Environment and Public Health	Investigative Techniques	1.42%
Health Care Quality, Access, and Evaluation	Investigative Techniques	1.26%
Environment and Public Health	Health Care Quality, Access, and Evaluation	1.18%
Diagnosis	Investigative Techniques	1.13%
Investigative Techniques	Persons	0.90%
Environment and Public Health	Persons	0.84%
Diagnosis	Environment and Public Health	0.82%
Health Care Quality, Access, and Evaluation	Persons	0.81%
Diagnosis	Persons	0.81%
Diagnosis	Health Care Quality, Access, and Evaluation	0.77%
Direct citation map		
Environment and Public Health	Investigative Techniques	1.44%
Diagnosis	Investigative Techniques	1.21%
Environment and Public Health	Health Care Quality, Access, and Evaluation	1.19%
Health Care Quality, Access, and Evaluation	Investigative Techniques	1.10%
Behavior and Behavior Mechanisms	Health Care Quality, Access, and Evaluation	1.10%
Behavior and Behavior Mechanisms	Psychological Phenomena	0.95%
Surgical Procedures, Operative	Therapeutics	0.94%
Investigative Techniques	Therapeutics	0.86%
Health Care Quality, Access, and Evaluation	Health Services Administration	0.76%
Behavior and Behavior Mechanisms	Environment and Public Health	0.76%

### Category C map

**Figure 3:**
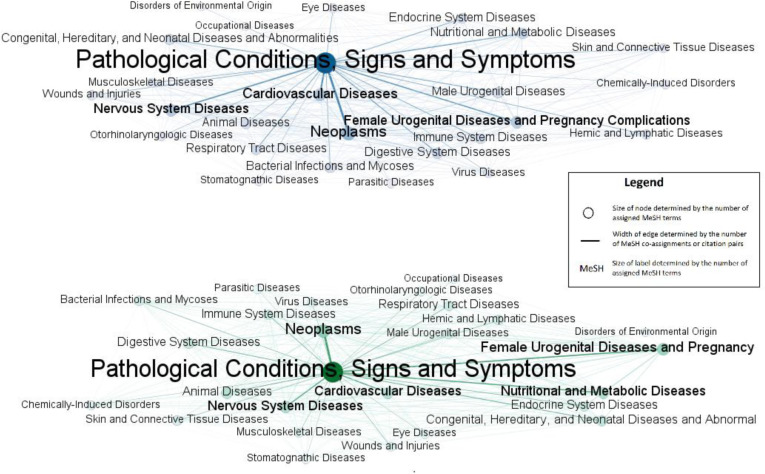
MeSH co-assignment map (upper) and MeSH direct citation map (lower) (Category C only)

When focusing on Category C MeSH terms, both the MeSH co-assignment map (upper, blue) and direct citation map (lower, green) presented a similar structure of biomedical science as shown in Figure 3. As same as the level 1 map, edge width is proportional to the number of co-assignments or citation pairs, and the node and label sizes are proportional to the number of assignments or citations. The shade of nodes/edges is also based on their size or width, as the color of large nodes or wide edges are darker. All diseases were connected to *Pathological Conditions, Signs and Symptoms,* which is the largest node and hub in both maps. Some nodes, such as *Neoplasms, Cardiovascular Diseases, Nervous System Diseases,* and *Female Urogenital Diseases and Pregnancy Complications,* had strong connections with *Pathological Conditions* and *Signs and Symptoms.*

## DISCUSSION

Our results show that MeSH co-assignments and MeSH citation pairs are two different approaches to measuring relationships among medical topics that can show the core structure of biomedical science [17]; MeSH co-assignment indicates relationships between topics in different categories (e.g., psychology and gender), whereas MeSH pairs of citing/cited articles indicate relationships between topics in the same or similar categories.

We found that co-assignment mapping and citation pair mapping yielded similar visualizations. MeSH co-assignments include all medical topics discussed in articles, leading to a high-density map containing more nodes and edges. By contrast, MeSH citation pairs indicate a connection between similar medical topics via a citation link, leading to a less dense map consisting of large nodes and wide edges.

Named Groups, most often assigned as nonmajor topics, frequently appeared in MeSH co-assignment maps but did not emerge in MeSH direct citation maps, as MeSH citation pairs only represent the relationship between two major medical topics from the respective citing article and cited reference. This difference was also seen in level 2 map; four strong edges between Person and Investigative Techniques, Environment and Public Health, Health Care Quality, Access, and Evaluation, and Diagnosis were observed in the MeSH co-assignment map but were hardly apparent in the MeSH direct citation map.

Such results do not mean that one map is more accurate or representative than the other; rather, they represent different angles of view of biomedical science. The MeSH co-assignment map shows relationships between topics in different categories, allowing us to observe whether a medical topic is related to a non-medical topic in Name Groups, Geographicals, or Publication Characteristics categories. On the other hand, the MeSH direct citation map shows relationships between topics within the same or similar categories, allowing us to view the core structure of biomedical science.

This study has some limitations. As we investigated all medical papers published in a single year, the maps do not show the evolution of biomedical sciences. In addition, cited references not indexed by WoS were excluded from this study; thus the Open Citation Collection recently introduced by National Institutes of Health [26] could be a better data source for future studies.

In addition, different visualization methodologies, affected by choice of software and algorithms, may also influence the appearance of the map. For example, Song and Chi found that VOSviewer and Citespace produced two different maps based on the same dataset due to their different default settings and clustering methods [27]. Also, in order to map all disciplines, citation-based maps have to normalize citation data since the citation rate varies among different disciplines, whereas subject headings co-assignment maps can visualize science without data normalization because the number of subject headings assigned is small [15].

In conclusion, the results of this study could form a foundation for future studies mapping science using subject headings. The comparison between the co-assignment map and direct citation map reflects the fundamental difference between two mapping techniques; subject headings show knowledge areas that must be learned together, whereas citation links express how one discipline draws knowledge from or builds upon another. Subject heading co-assignments could represent relationships between various research topics in different categories, not only from journal articles but also from non-fiction books and monographs, which broadens our understanding of the relationships between major sub-disciplines of science. In future work, we plan to validate this approach by producing maps based on other data collections.

## Data Availability

Data associated with this article are available in the Open Science Framework at https://osf.io/58rk6/.
